# Outcomes of Kissing Stent Technique for the Endovascular Treatment of Aorto-Iliac TASC C/D Lesions

**DOI:** 10.3390/jcm15187007

**Published:** 2026-09-10

**Authors:** Eleonora Acquisti, Rodolfo Pini, Enrico Gallitto, Mohammad Abualhin, Alessia Sonetto, Marcello Lodato, Stefania Caputo, Antonio Cappiello, Marco Mattiacci, Gianluca Faggioli, Mauro Gargiulo

**Affiliations:** 1 Vascular Surgery, Department of Medical and Surgical Sciences (DIMEC), University of Bologna, 40138 Bologna, Italy; rudypini@gmail.com (R.P.); enrico.gallitto@gmail.com (E.G.); marcello.lodato7@gmail.com (M.L.); caputostefania@hotmail.it (S.C.); antocapp9@gmail.com (A.C.); marco.mattiaccipg@gmail.com (M.M.); gianluca.faggioli@unibo.it (G.F.); mauro.gargiulo2@unibo.it (M.G.); 2 Vascular Surgery Unit, IRCCS Azienda Ospedaliero-Universitaria di Bologna, 40138 Bologna, Italy; m.abualhin@gmail.com (M.A.); ale.sonetto@gmail.com (A.S.)

**Keywords:** aortoiliac occlusive disease, hybrid revascularization, kissing stent, endovascular treatment, femoral endarterectomy, TASC II C/D lesions, peripheral arterial disease, aortic bifurcation

## Abstract

**Background:** Aorto-iliac Trans-Atlantic Inter-Society Consensus II (TASC II) C and D lesions with involvement of the aortic bifurcation are complex, and their endovascular treatment with kissing stenting can be technically demanding. The aim of this study is to analyze the outcomes, risk factors and follow-up of aorto-iliac revascularization through kissing stenting. **Methods:** A single-center, retrospective, observational study was performed including patients treated with aorto-iliac kissing stenting from 2016 to 2025 for TASC II C and D lesions. Pre-/peri- and post-operative data were prospectively collected and retrospectively analyzed. Aorto-iliac calcification consisted of calcific lesions involving more than 70% of the aortic circumference. Technical and clinical success, primary patency and reintervention rate were analyzed. **Results:** Overall, 123 patients were included, 48 (39%) female; mean age was 68 ± 7 years. Sixty-nine patients (56%) had critical limb-threatening ischemia, 46 (37%) diabetes mellitus, 5 (4%) end-stage chronic kidney disease in hemodialysis and 68 (55%) aortic calcification. Femoral accesses were percutaneous in 18 (15%) and surgical in 105 (85%) cases; additional brachial access was used in 49 (40%) patients. Simultaneous femoral endarterectomy was performed in 48 (39%) cases. Stentgrafts and bare metal stents were used in 32 (26%) and 91 (74%) cases, respectively. Technical success was 100%. Primary patency at 1, 3 and 5 years was 100%, 98 ± 3% and 94 ± 5%, respectively. At a mean follow up of 39 months, 6 (5%) stent thromboses occurred. Freedom from reintervention at 1, 3 and 5 years was 99%, 93% and 81%, respectively. Calcification was associated with a higher 3-year reintervention rate: 13% vs. 0%, *p* = 0.037. Brachial access, additional femoral endarterectomy and use of covered stents did not affect the reintervention rate. **Conclusions:** Kissing stent is an effective and safe technique to provide revascularization in aorto-iliac TASC C-D lesions with involvement of the aortic bifurcation, with good outcomes in terms of technical, clinical success and primary patency. It often needs an upper limb access and/or adjunctive procedures such as femoral endarterectomy. Presence of severe calcification is associated with a higher reintervention rate.

## 1. Introduction

Aorto-iliac occlusive disease (AIOD) is a progressive atherosclerotic condition commonly involving the iliac bifurcation affecting approximatively 3–10% of the general population. The prevalence is higher in young (<70 years old), smoking and dyslipidemic patients [1].

In 2007 Norgren et al. developed a classification based on the anatomical features of the disease, called the Trans-Atlantic Inter-Society Consensus II (TASC II) [2]. Types C and D represent the most challenging scenarios, particularly patterns showing extensive stenosis or occlusion of both iliac axes, or an aorto-iliac barrage.

Historically, open surgery has been considered the gold standard treatment for TASC C/D lesions, with excellent early and mid-term results in terms of patency, yet yielding a considerable rate of perioperative major complications [1]. The advent and evolution of endovascular techniques have shifted the treatment paradigm toward less invasive approaches [3]. Endovascular treatment can be performed with different procedures—CERAB (Covered Endovascular Reconstruction of the Aortic Bifurcation), barrel and kissing stenting (KS)—all of which are well-validated techniques.

The management of these lesions requires a balance between anatomical durability and the procedural burden of open reconstruction. Although aorto-bifemoral bypass remains a durable option, its invasiveness, the need for abdominal exposure, and the cardiovascular risk associated with major surgery may limit its use in patients with substantial comorbidity. Endovascular reconstruction has therefore gained importance, particularly when the disease can be crossed and adequately treated without compromising relevant pelvic or mesenteric collateral vessels. In this setting, the choice of reconstruction strategy depends not only on lesion length and occlusion pattern but also on the morphology of the aortic bifurcation, the degree of calcification, the condition of the common femoral arteries, and the extent of disease toward the external iliac arteries. CERAB and other anatomical reconstruction techniques may provide favorable geometric characteristics at the aortic bifurcation, whereas KS remains attractive because it is technically straightforward, widely available, and adaptable to different iliac anatomies [3,4,5].

**Figure 1:**
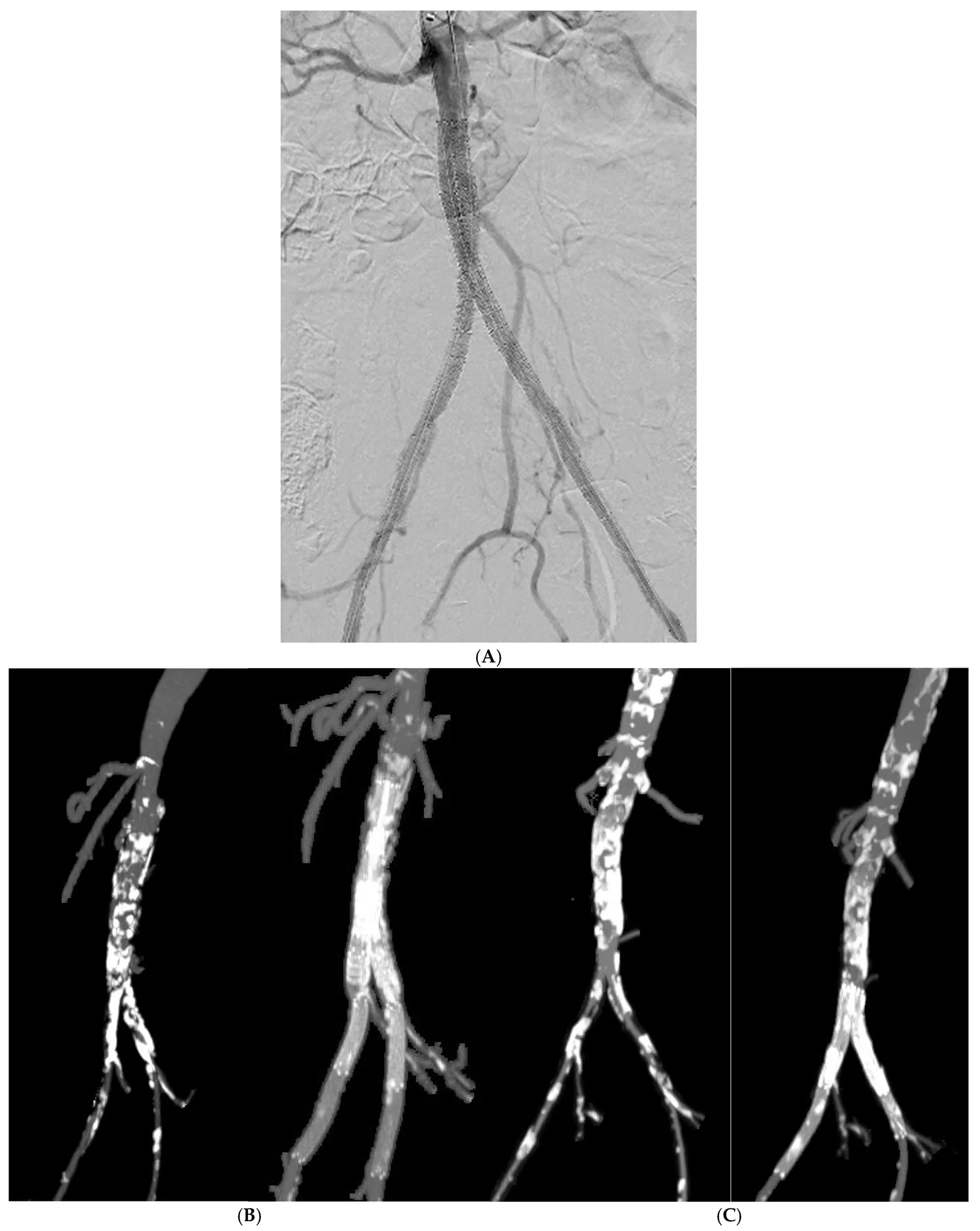
(**A**) The kissing stent technique consists in deploying two stents simultaneously in a kissing fashion, landing proximally in the distal aorta and distally in the common iliac arteries. Representative preoperative and postoperative CT angiographic reconstructions of the aorto-iliac bifurcation when using covered stents (**B**) and bare-metal stents (**C**), respectively.

Kissing stenting consists in the simultaneous bilateral deployment of stents at the aortic bifurcation for the treatment of lesions involving the aortic bifurcation and/or the common iliac arteries (Figure 1). In cases of challenging anatomy requiring upper extremity access, an antegrade recanalization may be required to increase the pushability. Nowadays KS is a widely adopted method, offering favorable technical success with reduced morbidity. On the other hand, supportive data concerning mid- and long-term results in terms of primary patency and reintervention, especially in complex anatomies, and correlation to specific risk factors have not been fully described.

The technical success of KS depends on obtaining stable wire access through both iliac axes and achieving adequate expansion and apposition of the two devices at the bifurcation. In heavily calcified disease, however, lesion crossing and stent expansion may be difficult, and residual plaque burden can influence the final geometry of the reconstructed bifurcation [6,7]. The proximal landing zone is also relevant because extending the devices too far into the distal aorta may compromise lumbar or inferior mesenteric collateral pathways, whereas insufficient proximal coverage may leave residual disease at the bifurcation. These considerations explain why device selection and access strategy are often individualized in complex TASC C/D lesions. Balloon-expandable stents provide controlled deployment and radial force at the aorto-common iliac segment, while self-expandable stents may be more adaptable when treatment extends distally into the external iliac artery. Covered stents may be useful in selected thrombotic or otherwise complex lesions, but their use must be balanced against the desire to preserve collateral branches.

Despite the growing body of evidence supporting an endovascular-first strategy, the long-term performance of KS in patients selected specifically for complex TASC II C/D lesions involving the aortic bifurcation remains incompletely characterized. Existing studies include heterogeneous anatomical patterns and technical approaches, and relatively few have focused on the relationship between severe aortic calcification, adjunctive access or femoral procedures, stent type, and subsequent reintervention [3,4,5,6,8]. Defining these associations may help identify patients in whom additional preparation or alternative reconstruction should be considered.

This study aims to evaluate the outcomes of kissing stenting for complex aorto-iliac TASC II C and D lesions and the impact of anatomical and technical factors on long-term outcomes.

## 2. Materials and Methods

*Study population:* A single-center, retrospective, observational study was conducted. Patients with TASC C/D lesions involving the aortic bifurcation treated with aorto-iliac kissing stenting for occlusive disease between January 2016 and October 2025 were included. The data were prospectively collected into a dedicated database and retrospectively analyzed.

Demographics, comorbidities and risk factors were evaluated as preoperative features. All patients underwent a preoperative Computed Tomography Angiography (CTA) scan and Doppler-ultrasound (DUS) examination to investigate the extension of the disease and the characteristics of the plaques. Aorto-iliac calcification was evaluated at axial and coronal projections of CTAs and assessed as presence of calcific lesions in more than 70% of the aortic wall, as previously reported [6].

*Procedural details:* Procedures were performed in a Philips hybrid operating room. Written consent from each patient was obtained before procedures. Percutaneous or cutdown accesses were based on the wall features of the common femoral arteries (calcification and/or presence of plaques). If a downward recanalization was necessary, because of challenging anatomy or technical failure from below, a left surgical brachial access was performed under general anesthesia. Lesions were crossed using standard guidewires and support catheters of appropriate size and length. Following lesion crossing, pre-dilatation was performed as needed, and two stents were deployed simultaneously in a kissing fashion, landing proximally in the distal aorta below the inferior mesenteric artery if possible, and distally in the common iliac arteries.

Stent choice (bare-metal or covered) was based on lesion morphology. Covered stents (CS) were selected for thrombotic lesions. Bare-metal stents (BMS) were used in all the remaining cases to preserve collateral pathways of lumbar arteries and inferior mesenteric artery, if the landing zone extended above it. BMSs included both balloon-expandable and self-expandable devices: balloon-expandable stents were preferentially deployed across the aorto-common iliac segment, whereas self-expandable stents were used when disease extended into the external iliac artery. Specific commercial device names and models were not prospectively recorded in the study database and therefore could not be analyzed separately.

Hemostasis was achieved with interrupted suture in surgically isolated femoral arteries, while percutaneous accesses were approached using the Perclose Prostyle suture-mediated closure system.

*Postoperative management:* Postoperative medical therapy was prescribed according to the anamnestic factors and the procedural details. Dual antiplatelet therapy (DAPT) was adopted as the standard postoperative regimen whenever clinically feasible for 1 to 6 months.

Postoperative surveillance included clinical examination, DUS at 6 months, CTA at 12 months and yearly DUS thereafter. On DUS, restenosis was considered as a >50% caliber reduction with flow demodulation and loss of the triphasic wave. When CTA was performed, restenosis was defined as >50% luminal diameter reduction on axial and multiplanar reconstructions.

*Endpoints:* Endpoints of the study were technical success (TS), clinical success (CS), primary patency (PP) and freedom from reintervention. TS was defined as patency with <30% residual stenosis and no other major complication, such as artery dissection or rupture or distal embolization. CS was defined by the improvement of at least one Rutherford category between preoperative and post-procedural clinical presentation. PP was defined as absence of >50% restenosis on imaging. Reintervention was defined as any additional endovascular or open procedure required to restore or maintain iliac/stent patency or to treat a procedure-related arterial complication. Access-site hematoma evacuation and groin wound dehiscence revision were classified as perioperative access-site complications and were not counted as reinterventions. Based on these definitions, risk factors for loss of patency and reintervention were searched among anatomic characteristics evaluated at CTA and adjunctive procedures, such as brachial access or femoral endarterectomy.

Statistical analysis: Continuous variables were described with median and interquartile range (IQR) and were compared by Mann–Whitney’s test. Categorical variables were reported as percentage and were compared using Fisher’s test. A value of *p* < 0.05 (two-tailed) was considered to be significant. Statistics were calculated with SPSS 23.0 software (SPSS Inc., Chicago, IL, USA).

## 3. Results

*Study population:* Between January 2016 and October 2025, 192 patients were treated in our center for both surgical and endovascular aorto-iliac revascularization procedures; among these, 123 patients underwent endovascular reconstruction of the aorto-iliac segment with the kissing stent technique for lesions TASC II C and D involving the aortic bifurcation. The mean age was 68 ± 7 years and 61% of them were male. The choice of a surgical revascularization was reserved to cases of aorto-iliac occlusion extending in the proximity of the renal arteries.

Demographics, preoperative cardiovascular risk factors and comorbidities are summarized in Table 1. Sixty-nine patients (56%) presented with critical limb-threatening ischemia. Smoking was the most prevalent cardiovascular risk factor (114 patients, 93%), followed by hypertension (105, 85%) and hyperlipidemia (82, 67%). Diabetes was present in 46 patients (37%), while chronic kidney disease greater than stage 3 was documented in 27 patients (22%), including 5 patients (4%) receiving hemodialysis. Coronary artery disease and chronic obstructive pulmonary disease were reported in 32 (26%) and 22 (18%) patients, respectively. Nine patients (7%) were obese and seven (6%) had atrial fibrillation. Internal iliac artery patency was preserved in 70 patients (57%). Severe aortic calcification, defined according to the prespecified CT criterion, was present in 68 patients (55%), while external iliac artery disease was identified in 73 patients (59%).

*Procedural details:* Cut-down femoral accesses were performed in the majority of cases (85%), and in 49 cases (40%) an additional surgical exposure of the proximal third of the arm was required. Accesses to brachial arteries were always surgical. Endarterectomy of the femoral bifurcation with patch angioplasty was performed in 39% of cases. Table 2 summarizes procedural details.

Femoral access was percutaneous in 18 patients (15%) and performed by surgical cut-down in 105 (85%), according to the presence of femoral wall calcification or plaque requiring direct control. An additional left brachial access was required in 49 patients (40%) when antegrade recanalization was considered necessary because of lesion complexity or failure of the femoral approach. Common femoral endarterectomy with patch angioplasty was performed in 48 patients (39%); the procedure was bilateral in 12 patients (10%). Additional treatment of the external iliac artery with PTA and/or stenting was required in 46 patients (37%), in cases with atherosclerotic disease extending beyond the common iliac segment. General anesthesia was used in 100 procedures (81%), whereas 23 patients (19%) were treated under local or locoregional anesthesia.

*Endpoints and statistical analysis:* Technical success was 100%. No perioperative major adverse events (acute myocardial infarction, acute respiratory failure, stroke) were observed. All patients (100%) presented improvement of the clinical presentation. Perioperative outcomes are reported in Table 3. Four patients (3%) developed stent thrombosis within 30 days from the procedure, and they were treated in an urgent setting with iliac recanalization and relining. Two patients (2%) returned to the operating room for hematomas and one (0.8%) for patch infection.

Bare-metal stents were used in 91 patients (74%), whereas covered stents were used in 32 patients (26%), mainly for lesions with a thrombotic component.

**Figure 2:**
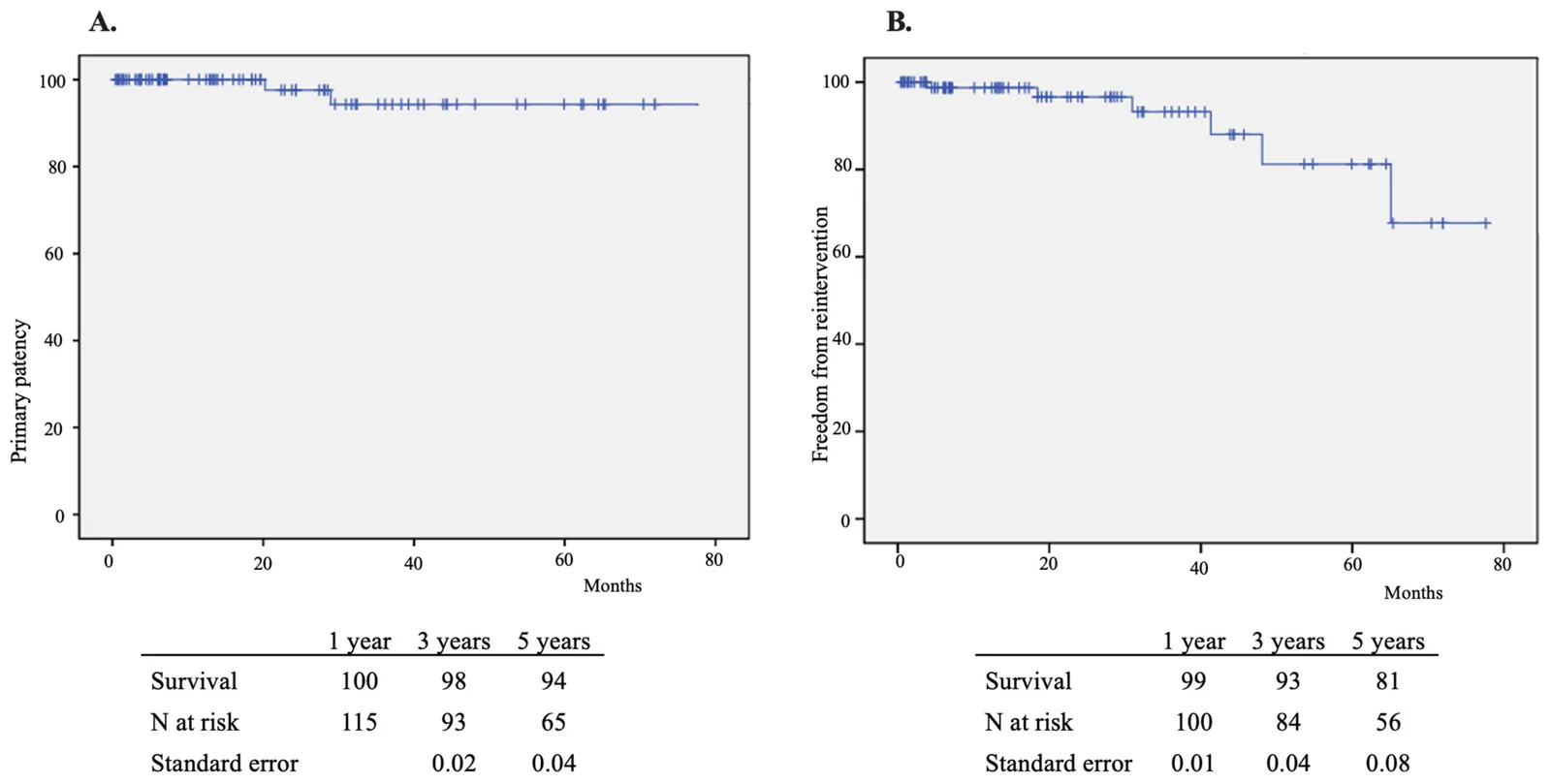
Overall five-year primary patency (**A**) and freedom from reintervention (**B**).

The mean follow-up was 39 months. Primary patency at 1, 3 and 5 years was 100%, 98 ± 3% and 94 ± 5%, respectively. Two patients (2%) experienced stent thrombosis during later follow-up (after the perioperative 30-day period). Freedom from reintervention at 1, 3 and 5 years was 99%, 93% and 81%, respectively (Figure 2). The Kaplan–Meier estimates account for censoring and therefore cannot be directly derived by dividing the number of observed reintervention events by the total cohort size.

Overall, seven patients met the prespecified reintervention endpoint during follow-up: four patients experienced early stent thrombosis within 30 days, two experienced late stent thrombosis after 30 days, and one required surgery for a delayed femoral patch infection. The two hematoma evacuations and the two groin wound dehiscence revisions were considered access-site complications and were therefore excluded from the reintervention endpoint. Thus, the number of reintervention procedures performed in the cohort is not equivalent to the cumulative number of patients experiencing the primary reintervention endpoint, and it should not be compared directly with the Kaplan–Meier estimate without accounting for follow-up time and censoring.

Of the two patients with late stent thrombosis, one underwent relining with CS of the right iliac axis one year after the main procedure; the other needed recanalization of both common iliac arteries and bilateral relining with two CSs. During the later follow-up, both remained free from restenosis or re-occlusion.

There was one case of femoral patch infection occurring eight months after the main procedure, which was treated with explant and ligation of the external iliac artery and, at a later stage, an axillo-femoral bypass. This event was counted as a reintervention because it required an additional surgical procedure for a procedure-related arterial complication. Two patients (2%) who developed dehiscence of the groin wound underwent surgical revision; these access-site revisions were not included in the reintervention endpoint.

**Figure 3:**
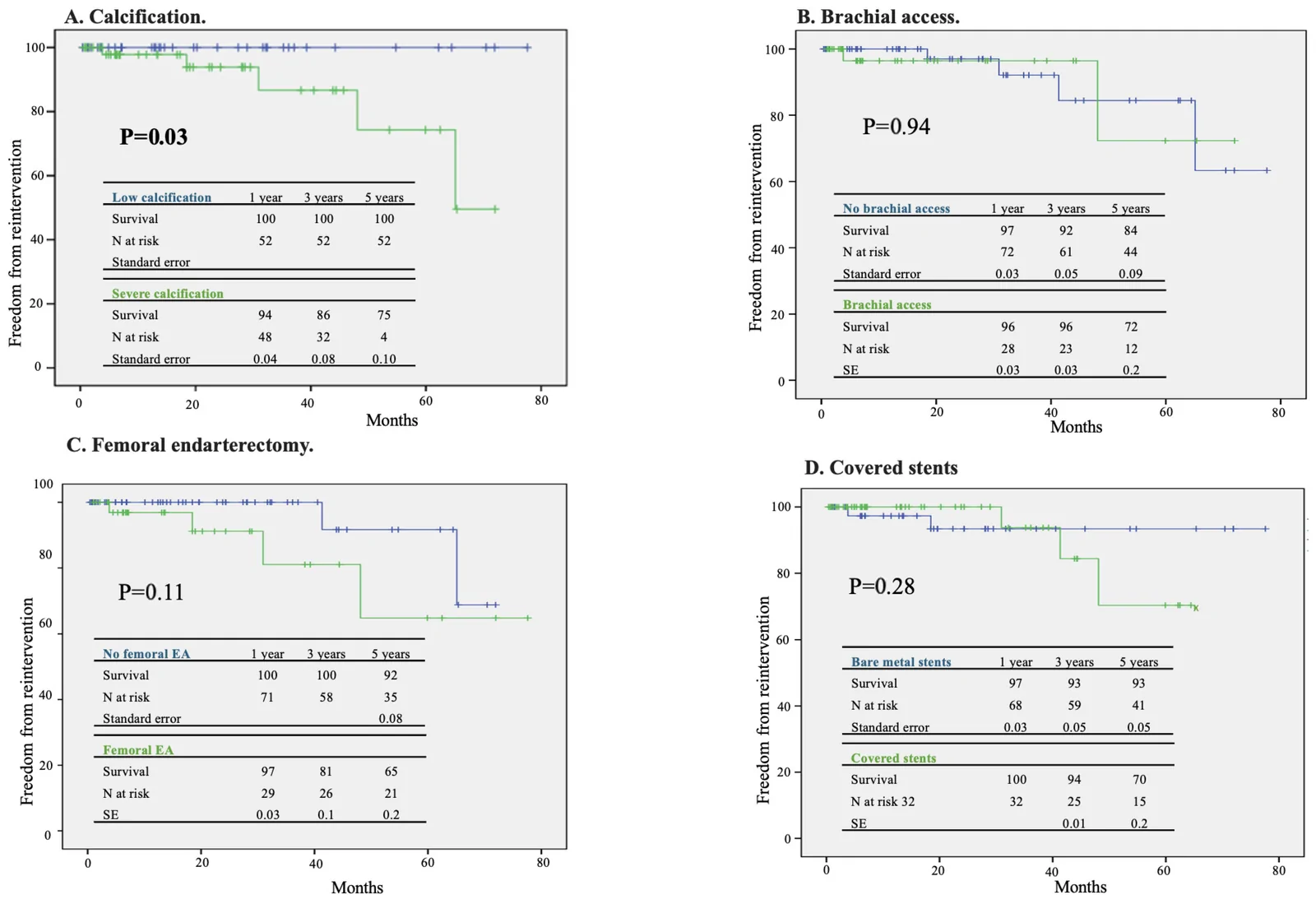
Estimated five-year freedom from reintervention according to aortic calcification (**A**), the use of a brachial access (**B**), adjunctive femoral endarterectomy (**C**) and covered stents (**D**). Numbers at risk reflect patients with available time-to-event data and may therefore differ from the baseline cohort size.

Aortic calcification was associated with a higher 3-year reintervention rate (13% vs. 0%, *p* = 0.037). Brachial access, additional femoral endarterectomy and use of CS did not affect the reintervention rate (Figure 3).

## 4. Discussion

Our experience with the kissing stent technique showed excellent technical success and durable patency outcomes, consistent with the recent literature findings. Technical success was as high as 100%, a result that aligns with or exceeds pooled data from major meta-analyses [3,4], where success rates for TASC C/D lesions consistently range between 95% and 98.5%. Comprehensive reviews of the endovascular-first approach for complex aorto-iliac disease, including the work by Jebbink et al. [5] in their systematic review, highlight its increasing safety and efficacy as a first-line treatment. Recent evidence from Piffaretti et al. (2022) [7] confirms that the progress of materials now allows the successful recanalization even of extensive aorto-iliac occlusions, significantly reducing the necessity of open surgical conversion.

The primary patency rates observed in our study—98% at 3 years and 94% at 5 years—ranks among the highest reported in the current literature. While systematic reviews report 2-to-3-year primary patency rates between 80% and 87% for complex lesions [9], our results reflect a high-performance cohort. Historical data and older series, such as those discussed by Shen et al. and Pulli (2015) [10,11], often questioned the long-term durability of endovascular techniques compared to open surgery. However, contemporary series show that while aorto-bifemoral bypass remains a benchmark for 10-year durability, the modern endovascular-first approach offers comparable mid-term outcomes with significantly lower perioperative morbidity [12].

The choice between CS and BMS remains a cornerstone of the clinical debate. Although our study did not find a statistically significant difference in reintervention rates between the two groups, broader literature increasingly favors CS for complex disease. The COBEST trial demonstrated that CS provides superior primary patency and a significantly lower rate of reintervention compared to BMS, especially in TASC C and D lesions [13]. In the present series, CS was selected for thrombotic lesions, whereas BMS was preferred in other anatomies, with balloon-expandable devices used primarily at the aorto-common iliac segment and self-expandable devices when the disease extended into the external iliac artery.

Clinical summaries, such as the iVS-2022 summary, and studies by Bontinis et al. highly recommend the use of CS to mitigate the risk of arterial rupture during the aggressive dilation required for calcified vessels [14]. Some authors [15,16] describe advanced anatomical reconstruction techniques, such as CERAB, stating they may offer superior flow dynamics compared to traditional KS, potentially reducing the risk of future restenosis. In our experience, the use of BMS was associated with outcomes comparable to CS in terms of patency and freedom from reintervention, although the present retrospective study was not designed as a non-inferiority comparison. Our approach to this pathology is based on the fact that the type of stent is chosen according to the plaque’s characteristics: in the presence of thrombotic aspects, CS is normally preferred. In all other cases, our aim is always to preserve lumbar, sacral and hypogastric arteries, so BMS is preferred whenever possible to avoid jeopardizing their patency.

A critical finding in our analysis was that severe aortic calcification (involving >70% of the wall) was associated with a higher risk of reintervention, with a 3-year rate of 13% vs. 0%. This is strongly supported in our previous work [6] and by studies of CT calcification patterns, which identify high calcific burden as a potential driver of incomplete stent expansion and subsequent flow disturbances. To address these anatomical challenges, Fazzini et al. [17] proposed the integration of adjunctive technologies such as intravascular lithotripsy (IVL) to prepare the vessel wall. Preparing calcified segments with IVL may facilitate better stent apposition and reduce the risk of elastic recoil or thrombotic complications.

The high incidence of brachial access (40%) and femoral endarterectomy (40%) in our cohort underscores the complexity of TASC C/D lesions. Current literature reinforces the value of a “hybrid” strategy where surgical common femoral artery reconstruction secures the outflow while proximal endovascular stenting treats the inflow [11,18]. As evidenced by our data, these adjunctive procedures do not negatively impact long-term outcomes but are essential tools for achieving technical success in hostile anatomies.

Despite the limitations of our retrospective design and the limited number of patients reaching a long-term follow-up, our findings support the endovascular-first strategy for TASC C and D lesions. The interpretation of long-term freedom from reintervention should take into account the progressive reduction in the number of patients at risk over time and the resulting influence of censoring on Kaplan–Meier estimates. Patient selection and precise preoperative planning remain the cornerstones for satisfactory outcomes. Continued research is essential to further define the optimal stent selection and post-procedural protocols, as suggested by recent meta-analytical data.

## 5. Conclusions

Our study confirms that the kissing stent technique for TASC C and D lesions is technically feasible, safe, and offers good mid-term durability. Technical success and primary patency are high, with low perioperative morbidity. Adjunctive procedures do not negatively impact outcomes, while severe calcification is associated with a higher risk of reintervention. The findings reinforce an endovascular-first strategy for TASC C and D lesions, provided that proper planning and appropriate adjuncts (e.g., brachial access and hybrid approach with femoral reconstruction) are used. Open surgery should be reserved for selected patients with highly calcified, coral reef-type lesions or after endovascular failure. Thus, patient selection and accurate preoperative planning remain cornerstones for satisfactory outcomes.

Further prospective, multicenter studies are warranted to define optimal stent choice, antiplatelet strategy, and treatment algorithm for different anatomical subgroups.

## Figures and Tables

**Table 1:** Demographics and comorbidities of the patients.

	*n*	%
Male sex	75	61
Hypertension	105	85
Smoking	114	93
Hyperlipidemia	82	67
Diabetes mellitus	46	37
Chronic obstructive pulmonary disease	22	18
Coronary artery disease	32	26
Chronic kidney disease (>stage 3) - Hemodialysis	27 5	22 4
Atrial fibrillation	7	6
Obesity	9	7
Critical limb-threatening ischemia	69	56
Rutherford cat. 3	54	44
Rutherford cat. 4	31	25
Rutherford cat. 5	32	26
Rutherford cat. 6	6	5
Internal iliac arteries patency	70	57
Aortic calcification	68	55
External iliac artery (EIA) disease	73	59
**Total**	**123**	**100**

**Table 2:** Procedural details.

	*n*	%
Percutaneous femoral access	18	15
Cutdown femoral access	105	85
Brachial access	49	40
Common femoral endarterectomy - Bilateral	48 12	39 10
Kissing stent with bare-metal stents	91	74
Kissing stent with covered stents	32	26
PTA/stenting external iliac artery	46	37
General anesthesia	100	81
Local/locoregional anesthesia	23	19
**Total**	**123**	**100**

**Table 3:** Perioperative and follow-up outcomes.

	*n*	%
Technical success	123	100
Clinical success	123	100
Acute myocardial infarction	0	0
Acute respiratory failure	0	0
Stroke	0	0
Acute kidney injury	1	0.8
Stent thrombosis < 30 days	4	3
Reintervention during follow-up (target/stent-related or procedure-related arterial complication)	7	6
**Total (patients)**	**123**	**100**
Iliac-axis thrombosis < 30 days	4	1.5
**Total (iliac arteries)**	**246**	**100**

## Data Availability

The data associated with the paper are available from the corresponding author upon reasonable request.

## Abbreviations

The following abbreviations are used in this manuscript:

TASC II	Trans-Atlantic Inter-Society Consensus II
CERAB	Covered Endovascular Reconstruction of the Aortic Bifurcation
CTA	Computed Tomography Angiography
DUS	Doppler ultrasound
CS	Covered stent
BMS	Bare-metal stent
DAPT	Dual antiplatelet therapy
TS	Technical success
PP	Primary patency
IQR	Interquartile range
IVL	Intravascular lithotripsy
KS	Kissing stent

## References

[1] Wang K., Cui Y., Zhang W., Jiang P., He J., Mei F. (2025). Treatment Options for TASC II C and D Aorto-iliac Occlusive Disease: A Systematic Review and Network Meta-analysis. J. Endovasc. Ther..

[2] Norgren L., Hiatt W.R., Dormandy J.A., Nehler M.R., Harris K.A., Fowkes F.G.R., TASC II Working Group (2007). Inter-Society Consensus for the Management of Peripheral Arterial Disease (TASC II). J. Vasc. Surg..

[3] Salem M., Hosny M.S., Francia F., Sallam M., Saratzis A., Saha P., Patel S., Abisi S., Zaye H. (2021). Management of Extensive Aorto-Iliac Disease: A Systematic Review and Meta-Analysis of 9319 Patients. Cardiovasc. Interv. Radiol..

[4] Groot Jebbink E., Holewijn S., Versluis M., Grimme F., Hinnen J.W., Sixt S., Angle J.F., Dorigo W., Reijnen M.M.P.J. (2019). Meta-analysis of Individual Patient Data After Kissing Stent Treatment for Aortoiliac Occlusive Disease. J. Endovasc. Ther..

[5] Groot Jebbink E., Holewijn S., Slump C.H., Lardenoije J.W., Reijnen M.M.P.J. (2017). Systematic Review of Results of Kissing Stents in the Treatment of Aortoiliac Occlusive Disease. Ann. Vasc. Surg..

[6] Sonetto A., Faggioli G., Pini R., Abualhin M., Goretti M., Fronterrè S., Pini A., Gargiulo M. (2021). Kissing Stent Technique for TASC C-D Lesions of Common Iliac Arteries: Clinical and Anatomical Predictors of Outcome. Ann. Vasc. Surg..

[7] Piffaretti G., Fargion A.T., Dorigo W., Pulli R., Ferri M., Antonello M., Bellosta R., Veraldi G., Benedetto F., Gargiulo M. (2022). Endovascular Reconstruction for Total Aorto-Iliac Occlusion. J. Endovasc. Ther..

[8] Dong X., Peng Z., Ren Y., Chen L., Sun T., Su Y., Liang H., Zheng C. (2023). Endovascular treatment of aorto-iliac occlusive disease with TASC II C and D lesions: 10 year’s experience of clinical technique. BMC Cardiovasc. Disord..

[9] Amanvermez Senarslan D., Yildırım F., Bayram B., Kurdal A.T., Tetik O. (2023). Results of endovascular treatments of Trans-Atlantic Inter-Society Consensus C or D aortoiliac occlusive disease involving the aortic bifurcation. SAGE Open Med..

[10] Shen C., Zhang Y., Qu C., Fang J., Liu X., Teng L. (2020). Outcomes of Total Aortoiliac Revascularization for TASC-II C&D Lesion with Kissing Self-Expanding Covered Stents. Ann. Vasc. Surg..

[11] Pulli R., Dorigo W., Fargion A., Angiletta D., Azas L., Pratesi G., Innocenti A.A., Pratesi C. (2015). Early and midterm results of kissing stent technique in the management of aortoiliac obstructive disease. Ann. Vasc. Surg..

[12] Pascarella L., Aboul Hosn M. (2018). Minimally Invasive Management of Severe Aortoiliac Occlusive Disease. J. Laparoendosc. Adv. Surg. Tech. A.

[13] Mwipatayi B.P., Thomas S., Wong J., Temple S.E.L., Vijayan V., Jackson M., Burrows S.A. (2011). A comparison of covered vs bare expandable stents for the treatment of aortoiliac occlusive disease. J. Vasc. Surg..

[14] Bontinis V., Bontinis A., Giannopoulos A., Manaki V., Kontes I., Rafailidis V., Antonopoulos C.N., Ktenidis K. (2024). Editor’s Choice—Covered Stents Versus Bare Metal Stents in the Treatment of Aorto-iliac Disease: A Systematic Review and Individual Participant Data Meta-analysis. Eur. J. Vasc. Endovasc. Surg..

[15] Reijnen M.M. (2020). Update on covered endovascular reconstruction of the aortic bifurcation. Vascular.

[16] Squizzato F., D’Oria M., Bozza R., Porcellato L., Grego F., Lepidi S. (2021). Propensity-Matched Comparison of Endovascular versus Open Reconstruction for TASC-II C/D AortoIliac Occlusive Disease. A Ten-Year Single-Center Experience with Self-Expanding Covered Stents. Ann. Vasc. Surg..

[17] Fazzini S., Pennetta F.F., Torsello G., Turriziani V., Vona S., Marchetti A.A., Ippoliti A., Austermann M., Bosiers M.J. (2025). Intravascular Iliac Artery Lithotripsy to Facilitate Aortic Endograft Delivery: Midterm Results of a Dual-Center Experience. J. Endovasc. Ther..

[18] Zacà S., Desantis C., Di Stefano L., Pulli R., Angiletta D. (2023). Outcomes of Endovascular Reconstructive Techniques in Trans-Atlantic Inter-Society Consensus II C-D Aortoiliac Lesions. Ann. Vasc. Surg..

